# Religious Attendance and Cancer Screening Behavior

**DOI:** 10.3389/fonc.2020.583925

**Published:** 2020-10-23

**Authors:** Benedikt Kretzler, Hans-Helmut König, André Hajek

**Affiliations:** Department of Health Economics and Health Services Research, Hamburg Center for Health Economics, University Medical Center Hamburg-Eppendorf, Hamburg, Germany

**Keywords:** cancer prevention, health care utilization, preventive medicine, cancer screening, religious affiliation, religion

## Abstract

**Background:** Cancer is one of the most important health problems worldwide. Preventive examinations proved to be effective in tackling that issue, but their degree of utilization is not adequate. Thus, research is making efforts to reveal its determinants. It has been shown that religion is associated with several health outcomes, so the aim of our study is to analyze the association between religious attendance and participation in cancer prevention.

**Methods:** Data are derived from the fifth wave of the German Aging Survey (DEAS), a nationally representative, prospective cohort study. Participants are community-dwelling Germans aged 40 years and older. Our main independent variable is the frequency of attendance in religious services, and the dependent variable is participation in cancer screening. As covariates, we include factors from all the dimensions of the Andersen behavioral health services utilization model. Multiple logistic regressions were used. In our sensitivity analysis, logistic regressions were performed stratified by religious group (Roman Catholic church, Protestant church, not belonging to any religious group).

**Results:** Our model shows that attendance in religious services once a week, one to three times a month, several times a year, or less often is significantly associated with an increased likelihood of participating in preventive cancer screening, more than never participating in religious services. Moreover, the sensitivity analysis reveals that all these associations remain significant for the Catholic subsample, but not for the Protestant or the non-religious group.

**Discussion:** This study finds a link between a higher frequency of attendance in religious services and an increased likelihood of participating in cancer screenings. This is important to address individuals at risk for underuse of cancer screenings.

## Introduction

Cancer is one of the most important health issues worldwide. In 2018, the International Agency for Research on Cancer counted 18.1 million new cases and 9.6 million deaths due to cancer. Moreover, prevalence has increased during the last years (1).

One effective instrument to reduce mortality is cancer screenings (2, 3). They facilitate early detection and treatment. Global guidelines have been defined by the World Health Organization (4). In Germany, the most frequently used prevention strategies are mammography and breast examination among women or colonoscopy and stool test among men (share of utilization in the respective age group) (5).

As preventive screenings prove to be effective in reducing cancer mortality, they often are supported by national health systems. For instance, German public health insurance covers the costs for screenings whose efficacy has been demonstrated. Despite these incentives, the degree of utilization is inadequate (6).

Due to this, research has made many efforts to reveal the determinants of cancer screening participation. The Andersen behavioral model of health services utilization, which is often used as a theoretical framework, distinguishes between predisposing (e.g., age), enabling (e.g., income), and need (e.g., chronic conditions) variables (7). It is assumed that these three factors independently influence health care utilization, and that need factors have the strongest effect (8).

Regarding predisposing components, higher education (9–13) and ethnicity (10, 12) are predictors of preventive cancer screening. Most of the studies also identify age as a significant correlate, but the association is found to be both positive (9, 10, 14–16) and negative (12, 13).

Considering enabling factors, the results are more distinct. Health insurance (10, 12, 14, 17, 18), income (10, 14), and socioeconomic status (19) are found to be associated with higher levels of screening participation.

Ultimately, among need factors, low health status (12, 14), the presence of current diseases (10), and a family history of cancer (16, 20) are associated with preventive cancer screenings.

Research has also considered the influence of religiousness on preventive cancer screening. However, most studies rely on specific samples, such as ethnic minorities. There are only a few studies that provide results for the association between religion and cancer screening that are based on a general population, mainly from North American countries.

Leyva et al. reveal that religious attendance is positively correlated with the use of various preventive cancer screenings. Some of these associations are moderated by social support (21). Benjamins reveals that religious salience is positively correlated with participating in cholesterol tests (22, 23), and O'Reilly shows that religiosity is associated with increased chances of attending breast examinations (24). On the other hand, Speed et al. do not find a significant association between religious engagement and cancer screenings (25).

Moreover, O'Reilly et al. detect that Catholics are significantly more likely to undergo breast cancer screening than denominational people (24). Zapka et al. find that Jewish women have a higher likelihood of having a mammogram than Catholic or Protestant women (26). Finally, Benjamins states that Evangelical Protestants may also be at risk of underusing preventive health services (27).

There are various hypotheses that try to explain these findings. First, religiosity or spirituality may help to develop a sensitivity toward one's own body and, therefore, support undertaking preventive health screenings. Previous research reveals a positive association between religiousness and healthy behaviors (28). Second, it may be possible that the embedment into a social group, such as a church community, provides a kind of social support that encourages people to follow a healthy lifestyle. For instance, a study from Krause, Shaw and Liang reveals that church-based support is associated with healthier behaviors among older African Americans (29). Furthermore, direct contact with health providers may also be achieved through church attendance. Both of these pathways rely on the assumption that religious practice promotes a healthy lifestyle. However, the second one also involves the role of social support in those associations. Apart from that, some other explanations may also be reasonable, such as religious people being more aware of their own health state in order to fulfill their responsibilities in their religious community.

As there are only a few studies that provide such evidence, the aim of our study is to examine whether religious attendance is linked to participation in preventive cancer screening, using a nationally representative sample. This may be important to determine individuals at risk for underuse of cancer screenings.

## Materials and Methods

### Sample

Data are derived from the German Aging Survey (DEAS). The DEAS is a nationally representative, prospective cohort study. All participants are community-dwelling individuals aged 40 years and older. Data collection occurs through standardized questionnaires carried out by trained interviewers.

The baseline sample of the DEAS took place in 1996. After that, follow-ups were conducted in 2002 (second wave), 2008 (third wave), 2011 (fourth wave), 2014 (fifth wave), and 2017 (sixth wave). Most of the follow-ups include both individuals that participate for the first time (cross-sectional sample) as well as individuals that have been questioned before (panel sample). The fourth and sixth waves only contain individuals that participated in a previous wave. In our study, we employed the fifth wave from 2014. It contains 10,324 individuals (interview). The subsequent questionnaire was answered by 7,952 individuals. In 2014, the response rate was 25% for the cross-sectional sample and 61% for the panel sample. For further information, please consider the report on data and methods of the DEAS (30, 31).

As we exclude participants whose data was missing for at least one of the variables considered in our regression models, our final sample size was *n* = 7,043. Written informed consent was given by all participants. The DEAS did not need an ethics vote because the criteria for obtaining it were not fulfilled.

### Dependent Variable

The dependent variable is participation in any kind of cancer screening. The corresponding question is “In the past years, did you regularly undergo early cancer screening?” (32). The participants answered “yes” or “no.” This is in accordance with other studies (33, 34).

### Independent Variables

Our main independent variable is attendance in religious services. We differ between six frequencies of occurrence (several times a week, once a week, one to three times a month, several times a year, less often, never).

Following the Andersen behavioral model of health services utilization, we also include several predisposing, enabling, and need variables (7). Predisposing factors are biological factors that are associated with health care utilization. Therefore, our regression model considers age and gender. Enabling factors take the influence of the social structure into account. We include education, graded according to the ISCED-classification (35), divided into three categories (low, middle, high), and monthly household net equivalence income.

Need factors represent the “direct” need for care from both objective and personal perspectives. The first need factor is physical functioning, instrumentalized by the correspondent subscale of the SF-36 [range from 0 to 100 with 0 as the worst and 100 as the best outcome. For further details, please see Ware and Sherbourne (36)]. We also add a sum score of physical illnesses. Therefore, the presence of 11 diseases, such as cardiac and circulatory disorders, is checked. Afterward, the number of conditions that occur within a participant is summed up to build the score. Finally, self-rated health (rated on a five-point scale with 1 as “very good,” 2 as “good,” 3 as “average,” 4 as “bad,” and 5 as “very bad”) is also used as a control variable.

### Statistical Analysis

Differences between the religious and non-religious groups are assessed by chi-square tests or ANOVAs. Logistic regression was performed, using participation in cancer screening as a dependent variable. Moreover, we performed a sensitivity analysis, conducting the logistic regression for the three most common categories of religious affiliations in Germany, which are belonging to the Catholic church, belonging to the Protestant church, and not belonging to any religious group (37). Significance level was set at 0.05. Analyses were carried out by Stata 14.0 (StataCorp, College Station, Texas, USA).

## Results

### Sample Characteristics

Table 1 shows the characteristics of our final analytical sample (*n* = 7,043) for the general population as well as for Catholics, Protestants, and people who do not belong to any religious group. Potential differences were analyzed using chi-square tests and ANOVAs as appropriate. Mean age is 64.19 (SD: 11.14), and age range is from 40 to 95. In total, 50.60% of the participants are female. Regarding education, 6.15% have a low education, 51.51% a middle one, and 42.34% are highly educated. The average monthly equivalent net income is 1949.77 EUR (SD: 1387.36 EUR). Mean physical functioning is 81.90 (SD: 21.73), mean count of physical illnesses is 2.59 (SD: 1.87), and mean self-rated health rating is 2.50 (SD: 0.83). More than one out of three participants had taken a cancer screening in the past years (34.50%).

**Table 1:** Sample characteristics for the individuals included in the regression analysis (*n* = 7,043).

	**Total sample**		**Catholics**		**Protestants**		**Undenominational**		***P*****-value**	
	**(*****n*** **=** **7,043)**		**(*****n*** **=** **1,845; 26.20%)**		**(*****n*** **=** **2,273; 32.27%)**		**(*****n*** **=** **2,723; 38.66%)**			
	**N/Mean**	**%/SD**	**N/Mean**	**%/SD**	**N/Mean**	**%/SD**	**N/Mean**	**%/SD**	
Age	64.19	11.14	63.99	11.07	65.75	11.31	63.22	10.86	*P* < 0.001
Sex									*P* < 0.001
Female	3,564	50.60%	968	52.47%	1,233	54.25%	1,264	46.42%	
Male	3,479	49.40%	877	47.53%	1,040	45.75%	1,459	53.58%	
Education (according to the ISCED-97 classification)									*P* < 0.001
Low	433	6.15%	168	9.11%	152	6.69%	74	2.72%	
Middle	3,628	51.51%	1,015	55.01%	1,183	52.05%	1,330	48.84%	
High	2,982	42.34%	662	35.88%	938	41.27%	1,319	48.44%	
Monthly equivalent income (in EUR)	1,949.77	1,387.36	2,037.44	1,491.28	1,944.63	1,177.17	1,919.40	1,473.31	*P* < 0.001
Physical functioning	81.90	22.73	82.62	21.65	80.66	23.62	82.65	22.57	*P* < 0.05
Number of physical illnesses (from 0 to 11)	2.59	1.87	2.62	1.86	2.63	1.88	2.50	1.85	*P* < 0.01
Self-rated health (from 1 = “very good” to 5 = “very bad”)	2.50	0.83	2.46	0.80	2.50	0.81	2.52	0.86	*P* < 0.05
Attendance in religious services									*P* < 0.001
Several times a week	126	1.79%	64	3.47%	18	0.79%	1	0.04%	
Once a week	455	6.46%	319	17.29%	89	3.92%	2	0.07%	
One to three times a month	560	7.95%	257	13.93%	277	12.19%	6	0.22%	
Several times a year	1,158	16.44%	452	24.50%	602	26.48%	78	2.86%	
Less often	2,370	33.65%	594	32.20%	1,041	45.80%	691	25.38%	
Never	2,374	33.71%	159	8.62%	246	10.82%	1,945	71.43%	
Cancer screening									
No	2,430	34.50%	640	34.69%	739	32.51%	965	35.44%	
Yes	4,613	65.50%	1,205	65.31%	1,534	67.49%	1,758	64.56%	*P* < 0.001

Considering religious attendance, approximately one out of six individuals regularly participated in religious convocations (several times per week: 1.79%; once a week: 6.46%; one to three times a month: 7.95%). Half of the individuals participated either several times a year (16.44%) or less often (33.65%). One third (33.56%) never went to religious services.

The most common religious denomination was Protestantism (32.27%), followed by Catholicism (26.20%). A small proportion had another religious belief (1.88%). However, the majority did not belong to any religious group (37.92%).

### Regression Analysis

The results of our main regression analysis are displayed in Table 2. Our dependent variable is participation in any cancer screening in the past years.

**Table 2:** Determinants of participation in cancer screening (*n* = 7,043).

**Independent variables**	**Cancer screening**	***P* < |z|**
	**(0 = no; 1 = yes)**	
Age	0.90 (0.62–1.31)	0.55
Sex (ref. male):		
Female	2.20 (1.98–2.45)	*P* < 0.001
Education (ref.: low, according to the ISCED-97 classification)		
Middle	1.67 (1.35–2.07)	*P* < 0.001
High	2.04 (1.63–2.54)	*P* < 0.001
Monthly equivalent income (in EUR)	1.00 (1.00–1.00)	*P* < 0.05
Physical functioning	1.01 (1.00–1.01)	*P* < 0.001
Number of physical illnesses (from 0 to 11)	1.08 (1.05–1.12)	*P* < 0.001
Self-rated health (from 1 = “very good” to 5 = “very bad”)	1.03 (0.96–1.12)	0.38
Attendance in religious services (ref. never):		
Several times a week	0.90 (0.62–1.31)	0.58
Once a week	1.31 (1.05–1.64)	*P* < 0.05
One to three times a month	1.31 (1.07–1.60)	*P* < 0.05
Several times a year	1.19 (1.02–1.39)	*P* < 0.05
Less often	1.29 (1.14–1.46)	*P* < 0.001
Pseudo *R*^2^	0.034	

Results of multiple logistic regressions.

*Odds ratios are displayed; 95% CI in parentheses*.

Compared to individuals who do not attend in religious services (“never”), going to religious services once a week (OR = 1.31, 95% CI: 1.05–1.64), one to three times a month (OR = 1.31, 95% CI: 1.07–1.60), several times a year (OR = 1.19, 95% CI: 1.02–1.39), or less often (OR = 1.29, 95% CI: 1.14–1.46) are associated with an increased likelihood of participation in cancer screening.

Nearly all our control variables from the Andersen behavior health service utilization model are significant correlates. Female sex (OR = 2.20, 95% CI: 1.98–2.45), middle (OR = 1.67, 95% CI: 1.35–2.07) or high (OR = 2.04, 95% CI: 1.63–2.54) education, monthly equivalent income (OR = 1.00, 95% CI: 1.00–1.00), physical functioning (OR = 1.01, 95% CI: 1.00–1.01), and the number of physical illnesses (OR = 1.08, 95% CI: 1.04–1.12) are all associated with an increased likelihood of taking a cancer screening. However, age (OR = 1.00, 95% CI: 1.00–1.01) and self-rated health (OR = 1.03, 95% CI: 0.96–1.12) are not associated with the outcome measure.

### Sensitivity Analysis

To shed more light onto this link, we also conducted a sensitivity analysis. More precisely, we performed the regression analysis for the three most common religious denominations in Germany: Catholicism, Protestantism, and not belonging to any religious group. Concerning the latter group, a survey from the Swiss Federal Statistical Office shows that nearly 40% of those individuals have visited a worship in the past 12 months (38). Results are tabulated in Table 3.

**Table 3:** Determinants of participation in cancer screening.

	**Catholic (*****n*** **=** **1,845)**		**Protestant (*****n*** **=** **2,273)**		**Undenominational (*****n*** **=** **2,723)**	
	**Cancer screening**	***P* < |z|**	**Cancer screening**	***P* < |z|**	**Cancer screening**	***P* < |z|**
	**(0 = no; 1 = yes)**		**(0 = no; 1 = yes)**		**(0 = no; 1 = yes)**	
Age	0.99 (0.98–1.00)	*P* = 0.32	1.00 (0.99–1.01)	*P* = 0.62	1.01 (1.00–1.02)	*P* = 0.08
Sex (ref.: male)						
Female	2.04 (1.65–2.51)	*P* < 0.001	2.11 (1.75–2.55)	*P* < 0.001	2.26 (1.91–2.67)	*P* < 0.001
Education (ref. low, according to the ISCED-97 classification):						
Middle	1.46 (1.02–2.08)	*P* < 0.05	1.91 (1.33–2.75)	*P* < 0.001	1.53 (0.94–2.49)	*P* = 0.09
High	1.29 (0.87–1.90)	*P* = 0.20	2.26 (1.54–3.31)	*P* < 0.001	2.22 (1.35–3.65)	*P* < 0.05
Monthly equivalent income (in EUR)	1.00 (1.00–1.00)	*P* < 0.05	1.00 (1.00–1.00)	*P* = 0.63	1.00 (1.00–1.00)	*P* = 0.27
Physical functioning	1.00 (1.00–1.00)	*P* = 0.24	1.01 (1.01–1.02)	*P* < 0.001	1.01 (1.00–1.01)	*P* < 0.05
Number of physical illnesses (from 0 to 11)	1.04 (0.97–1.11)	*P* = 0.25	1.11 (1.05–1.17)	*P* < 0.01	1.09 (1.03–1.14)	*P* < 0.01
Self-rated health (from 1 = “very good” to 5 = “very bad”)	1.04 (0.89–1.21)	*P* = 0.62	1.11 (0.97–1.27)	*P* = 0.14	0.98 (0.87–1.11)	*P* = 0.78
Attendance in religious services (ref. never):						
Several times a week	1.28 (0.70–2.34)	*P* = 0.42	1.20 (0.41–3.56)	*P* = 0.74		
Once a week	2.37 (1.58–3.57)	*P* < 0.001	0.99 (0.59–1.67)	*P* = 0.97	0.69 (0.04–11.38)	*P* = 0.80
One to three times a month	2.37 (1.55–3.63)	*P* < 0.001	1.11 (0.77–1.61)	*P* = 0.57	1.60 (0.27–9.54)	*P* = 0.61
Several times a year	1.77 (1.22–2.57)	*P* < 0.01	1.19 (0.87–1.63)	*P* = 0.29	1.04 (0.64–1.70)	*P* = 0.86
Less often	1.51 (1.06–2.17)	*P* < 0.01	1.34 (1.00–1.80)	*P* = 0.05	1.37 (1.13–1.67)	*P* < 0.01
Pseudo *R*^2^	0.039		0.035		0.040	

Results of multiple logistic regressions stratified by religion.

*Odds ratios are displayed; 95% CI in parentheses*.

As to the results of the Catholic subsample (*n* = 1,845), attending religious services once a week (OR = 2.37, 95% CI: 1.58–3.57), one to three times a month (OR = 2.37, 95% CI: 1.55–3.63), several times a year (OR = 1.77, 95% CI: 1.22–2.57), and less often (OR = 1.51, 95% CI: 1.06–2.17) compared to individuals who never attend religious services, are associated with an increased likelihood of cancer screening attendance. Only going to religious assemblies several times a week (OR = 1.28, 95% CI: 0.70–2.34) was not.

In terms of control variables, female sex (OR = 2.04, 95% CI: 1.65–2.51), a middle educational level (OR = 1.29, 95% CI: 0.87–1.90)—compared to a low educational level—and a higher monthly equivalent income (OR = 1.00, 95% CI: 1.00–1.00) are associated with an increased likelihood of taking cancer screenings.

In consideration of the Protestant subset (*n* = 2,273), none of the different frequencies of religious attendance are significantly associated with cancer screening (several times a week: OR = 1.20, 95% CI: 0.41–3.56; once a week: OR = 0.99, 95% CI: 0.59–1.67; one to three times a month: OR = 1.11, 95% CI: 0.77–1.61; several times a year: OR = 1.19, 95% CI: 0.87–1.63; less often: OR = 1.34, 95% CI: 1.00–1.80).

Most of the control variables are significantly linked to cancer screening. Being female (OR = 2.11, 95% CI: 1.75–2.55), middle (OR = 1.91, 95% CI: 1.33–2.75) or high (OR = 2.26, 95% CI: 1.54–3.31) education, physical functioning (OR = 1.01, 95% CI: 1.01–1.02), and the number of physical illnesses (OR = 1.11, 95% CI: 1.05–1.17) are correlated with an increased probability of cancer screenings.

Considering people who do not belong to any religious denomination, we exclude individuals who attend religious services several times per week as only one individual was concerned.

Only attending religious services “less often” compared to “never” had a significant link to cancer screening participation (OR = 1.37, 95% CI: 1.13–1.67); the other frequencies did not (once a week: OR = 0.69, 95% CI: 0.04–11.38; one to three times a month: OR = 1.60, 95% CI: 0.27–9.54; several times a year: OR = 1.04, 95% CI: 0.64–1.70).

Among the control variables, female sex (OR = 2.26, 95% CI: 1.91–2.67), high education (OR = 2.22, 95% CI: 1.35–3.65), physical functioning (OR = 1.00, 95% CI: 1.00–1.01), and the number of physical illnesses (OR = 1.09, 95% CI: 1.03–1.14) are significant correlates of an increased cancer screening participation.

Additionally, we checked whether the religious group moderates the association between attendance in religious services once a week and the likelihood of cancer screenings. Actually, there are significant differences between Catholic and Protestants (*p* < 0.05) as well as people who do not belong to any religious denomination (*p* < 0.01) with regard to the link between going to religious services once a week and the likelihood of cancer screenings, whereas other interaction terms mainly do not achieve statistical significance.

## Discussion

### Main Findings

Employing a large, nationally representative sample, the study goal was to determine the association between religious attendance and the undertaking of preventive cancer screenings. Moreover, we aimed to determine this link among different religious groups.

Multiple logistic regressions show that more frequent religious attendance is associated with increased levels of cancer screening participation. Furthermore, these findings only remain significant for the Catholic subsample and not for the Protestant one. Moreover, visiting worship less often than several times a year is significantly associated with an increased probability among individuals who do not belong to any religious group.

### Relation to Previous Research and Possible Explanations

In total, there are only a few studies that estimate the association between religiosity and use of preventive cancer screening. We build upon this knowledge and test this association in Germany. Beyond that, we extend our current knowledge by showing that this relation is mainly due to the Catholic participants in our sample.

We showed that religious attendance increases the likelihood of the undertaking of a cancer screening among middle-aged and older individuals in Germany. This is in accordance with the majority of existing studies (21–24). Moreover, the same pattern occurs when researchers test the relationship between religious attendance and the utilization of various kinds of preventive health care, such as physical examinations (39). These findings might be explained by claiming that religious people, more than the average, tend to take care of their body. Recently, a large population survey among various European countries revealed that individuals with increased religious engagement have a lower probability of smoking, alcohol consumption, physical inactivity, and doing no vigorous physical activity (28). However, one's health is not only promoted by avoiding damaging behaviors, but also by the undertaking of healthy habits. A study from Hill et al. reveals that religiousness is positively related to a score that contains healthy activities, such as physical exercise or vitamin consumption (40). Thus, the undertaking of preventive screenings may also be a part of such a healthy lifestyle.

One might also argue that religiousness has a social dimension, as attending religious services brings you together with many other people who have the same interests. Lee et al. reveal that a bigger social network is positively associated with participation in gastric cancer screening (41), and Bremer et al. show that informal support increases the likelihood of cancer screening, also using the German Aging Survey (42).

Finally, people who often attend worship may have certain characteristics that promote their regular attendance at early checkups. For instance, going to church regularly might be associated with an importance of schedules in one's life, which also affects regular cancer screenings. Moreover, previous research reveals that religiousness is associated with agreeableness, extraversion, and conscientiousness (43). Particularly, the latter was found to be linked with higher participation in cancer screenings as well (44).

Indeed, this does not explain the nonsignificant link between attending religious services several times per week and participation in preventive cancer screening that is found in our study. Using a qualitative approach among Hispanic Catholic churchgoers living in Massachusetts, Leyva et al. find that they partially have quite fatalistic beliefs about cancer and cancer prevention (45). A possible explanation may be that a fair share of people who visit worship in such a high frequency may tend toward some fundamentalist beliefs and, therefore, do not (only) rely on medical interventions to reduce cancer risk.

Regarding the differences between Catholics, Protestants, and those who are not members of any religious group, previous research reveals that Protestants are more likely to undergo breast examinations (24) as well as preventive cancer screening in general (46) for Northern Irish women. A possible explanation might be that a higher importance of the religious denomination in Northern Ireland, as Christians and Protestants were standing in conflict against each other not a long time ago, may have led to stronger differences between these subgroups. In Germany, confession hardly plays a role in one's daily life or life choices (47).

It is worth mentioning that attending religious services is a highly significant variable among Catholics although it is not significant among Protestants in our study. In turn, participating in religious events less often than several times a year is a significant correlate among both Protestants and people without any religious denomination; these two regressions seem to be more similar than the Catholic and the Protestant ones. As Catholics in our sample are more likely to regularly attend religious services than Protestants, that might be due to a higher importance of religion in their daily routine. Thus, the pathways that we describe above, such as taking care of one's body and soul or receiving social support, might be more valid for Catholics than for Protestants. Nevertheless, future research is required to explore the underlying mechanisms.

### Strengths and Limitations

Unlike other studies that almost exclusively focus on the link between religion and cancer screening in North American countries, this is the first study that derives evidence on the association between religion and preventive cancer screening for a country that is member of the European Union.

Moreover, it is one of only a few studies that distinguish between several religious (or non-religious) groups.

In addition, we must mention two weaknesses that are linked to our sample. The question “In the past years, did you regularly undergo early cancer screening?” has a high face validity; however, it does not differentiate between any kinds of cancer prevention. In addition, the DEAS has a minor selection bias (31), which might slightly affect the representativeness of our sample.

## Conclusion

We find that higher religious attendance is significantly associated with increased likelihood of preventive cancer screening participation. This might be beneficial for the development of cancer screening programs as it defines some population groups that are at risk of underuse. In addition, considering the decreasing value of religiousness in our times, there could be a need for additional efforts to maintain or even increase the screening participation rates.

## Data Availability Statement

Publicly available datasets were analyzed in this study. This data can be found at: https://www.dza.de/forschung/deas/datennutzung.html.

## Ethics Statement

Ethical review and approval was not required for the study on human participants in accordance with the local legislation and institutional requirements. The patients/participants provided their written informed consent to participate in this study.

## Author Contributions

BK, H-HK, and AH: design and concept of analyses, preparation of data, statistical analysis, interpretation of data, and preparing of the manuscript. All authors critically reviewed the manuscript, provided significant editing of the article, and approved the final manuscript.

## Conflict of Interest

The authors declare that the research was conducted in the absence of any commercial or financial relationships that could be construed as a potential conflict of interest.
